# 

**Published:** 1898-12

**Authors:** 


### CONTENTS.

**ORIGINAL COMMUNICATIONS.** PAGE

Independent Journalism from a Dental Stand-Point. By GEORGE S. ALLAN, A.B., D.D.S., New York City . . . 761

Artificial Dentures in their Relation to the Speaking or the Singing Voice. By DWIGHT L. HUBBARD, M.D., New York . . . 774

Taveau's Pâte d'Argent not the First Amalgam. By WILLIAM H. TRUEMAN, D.D.S., Philadelphia . . . 779

The First Meeting with Patients. By DWIGHT M. CLAPP, D.M.D. . . . 786

Unite the Forces. By GEORGE H. CHANCE, D.D.S., M.D., Portland, Ore. . . . 790

Reminiscences of a European Trip to the International Medical Congress at Moscow, Russia, August 19 to 26, 1897, as a Delegate from the American Medical Association and the Odontological Society of Pennsylvania. (*Concluded.*) By W. G. A. BONWILL, D.D.S., Philadelphia . . . 794

**ABSTRACTS AND TRANSLATIONS.**

A Simple Saliva Ejector. By J. J. H. SANDERS, L.D.S.I. . . . 801

**REPORTS OF SOCIETY MEETINGS.**

National Dental Association . . . 804

**EDITORIAL.**

The Last Month of the Year . . . 817

Special Notice . . . 819

**BIBLIOGRAPHY** . . . 819

**DOMESTIC CORRESPONDENCE.**

Legitimate Dental Practice hampered by Law . . . 821

**NOTES AND COMMENTS** . . . 822

**CURRENT NEWS.**

Pennsylvania State Board of Dental Examiners . . . 824

New York Odontological Society . . . 825

National School of Dental Technics . . . 826

